# Genome-Wide Analyses Reveal the Genetic Architecture and Candidate Genes of Indicine, Taurine, Synthetic Crossbreds, and Locally Adapted Cattle in Brazil

**DOI:** 10.3389/fgene.2021.702822

**Published:** 2021-07-27

**Authors:** Lucas Lima Verardo, Fabyano Fonseca e Silva, Marco Antonio Machado, João Cláudio do Carmo Panetto, Daniele Ribeiro de Lima Reis Faza, Pamela Itajara Otto, Luciana Correia de Almeida Regitano, Luiz Otávio Campos da Silva, Andréa Alves do Egito, Maria do Socorro Maués Albuquerque, Ricardo Zanella, Marcos Vinicius Gualberto Barbosa da Silva

**Affiliations:** ^1^Animal Breeding Lab, Department of Animal Science, Universidade Federal dos Vales do Jequitinhonha e Mucuri, Diamantina, Brazil; ^2^Department of Animal Science, Universidade Federal de Viçosa, Viçosa, Brazil; ^3^Embrapa Gado de Leite, Juiz de Fora, Brazil; ^4^Department of Animal Science, Universidade Federal de Santa Maria, Santa Maria, Brazil; ^5^Embrapa Pecuária Sudeste, São Carlos, Brazil; ^6^Embrapa Gado de Corte, Campo Grande, Brazil; ^7^Embrapa Recursos Genéticos e Biotecnologia, Parque Estação Biológica, PqEB, Brasília, Brazil; ^8^Department of Veterinary Medicine, Universidade de Passo Fundo, Passo Fundo, Brazil

**Keywords:** runs of homozygosity, effective population size, linkage disequilibrium, homozygosity islands, milk production, behavior, rusticity, fertility

## Abstract

Cattle population history, breeding systems, and geographic subdivision may be reflected in runs of homozygosity (ROH), effective population size (*N*_e_), and linkage disequilibrium (LD) patterns. Thus, the assessment of this information has become essential to the implementation of genomic selection on purebred and crossbred cattle breeding programs. In this way, we assessed the genotype of 19 cattle breeds raised in Brazil belonging to taurine, indicine, synthetic crossbreds, and Iberian-derived locally adapted ancestries to evaluate the overall LD decay patterns, *N*_e_, ROH, and breed composition. We were able to obtain a general overview of the genomic architecture of cattle breeds currently raised in Brazil and other tropical countries. We found that, among the evaluated breeds, different marker densities should be used to improve the genomic prediction accuracy and power of genome-wide association studies. Breeds showing low *N*_e_ values indicate a recent inbreeding, also reflected by the occurrence of longer ROH, which demand special attention in the matting schemes to avoid extensive inbreeding. Candidate genes (e.g., *ABCA7*, *PENK*, *SPP1*, *IFNAR1*, *IFNAR2*, *SPEF2*, *PRLR*, *LRRTM1*, and *LRRTM4*) located in the identified ROH islands were evaluated, highlighting biological processes involved with milk production, behavior, rusticity, and fertility. Furthermore, we were successful in obtaining the breed composition regarding the taurine and indicine composition using single-nucleotide polymorphism (SNP) data. Our results were able to observe in detail the genomic backgrounds that are present in each breed and allowed to better understand the various contributions of ancestor breeds to the modern breed composition to the Brazilian cattle.

## Introduction

Cattle domestication has generated many differentiated phenotypes that evolved into distinct breeds scattered across the globe. Evidence based on mitochondrial DNA (mtDNA) data indicates that divergence between taurine and indicine subspecies occurred approximately 10,000 years ago, long before domestication (Loftus et al., 1994). It is estimated that taurine cattle emerged in the Fertile Crescent region spanning modern-day Iraq, Syria, Lebanon, Israel, Palestine, Jordan, the northeast and Nile valley regions of Egypt, the south-eastern region of Turkey, and the western fringes of Iran. On the other hand, indicine cattle emerged in the Indus Valley, including northeast Afghanistan, including a great part of Pakistan, and western and northwestern India (Ajomone-Marsan et al., 2010). Some specific traits that shaped the domesticated bovine species took place long after domestication started, and artificial selection brought even more complexity and diversity to cattle subspecies in accordance with their evolving environment.

In Brazil, the first taurine animals were introduced by the European conquerors between the sixteenth and seventeenth centuries to be used for food, leather, and animal traction. After several generations of random crossings in distinct and variable ecosystems throughout the country, these animals became adapted to a wide range of environments and showed different levels of improved fitness to local conditions. They become recognized as Iberian-derived locally adapted breeds (Egito et al., 2007; McManus et al., 2009), such as the Caracu lineages (CaracuCaldeano for dairy purpose and Caracu for beef), CriouloLageano, CurraleiroPé-Duro, and CriouloLageano do RS.

In addition to the taurine breeds, indicine cattle were brought to Brazil in the nineteenth century (Medrado, 2018). Since then, indicine breeds (e.g., Nelore and Gir) have been extensively used to increase the production and robustness of the cattle herds throughout continental Brazil. The indicine breeds have also been successfully used to the generation of crossbreds and synthetic breeds in Brazil such as Girolando dairy cattle and Canchim beef cattle (Buzanskas et al., 2015). Thus, Brazil currently displays four genetic groups of cattle: taurine, indicine, locally adapted, and synthetic crossbreds.

Population history, breeding systems, and geographic subdivisions of the Brazilian cattle may be accessed through runs of homozygosity (ROH) and linkage disequilibrium (LD) patterns throughout the genome. Runs of homozygosity are continuous homozygous segments of DNA (Gibson et al., 2006), while LD is a nonrandom association between alleles at different loci (Ardlie et al., 2002). The calculation of these parameters is essential to perform genome-wide association studies (GWAS) and also to implement genomic selection (GS) in cattle breeds. Several studies have shown that genomic associations and estimated breeding values (EBVs) are affected by the LD pattern among populations (Meuwissen et al., 2001; Hayes et al., 2009; Marigorta and Navarro, 2013; Veroneze et al., 2014). In addition, Luan et al. (2014) showed that predicting genomic EBV based on ROH resulted in more accurate estimates when compared to EBV generated through linkage analysis relationships. Runs of homozygosity information also allows the identification of islands of homozygosity shared among individuals, which may be the result of selection events (Zhang et al., 2015). Thus, the knowledge of LD and ROH patterns in purebred and crossbred cattle has become essential for designing efficient genomic selection programs.

In this way, we assessed genomic information of 19 cattle breeds representative of four groups: (1) taurine (Angus, Brown Swiss, Holstein, and Jersey); (2) indicine (Brahman, Gir, Indubrasil, Nelore, Sindhi, and Tabapuã); (3) synthetic crossbreds (Canchim and Girolando); and (4) Iberian-derived locally adapted breeds (CriouloLageano, CurraleiroPé-Duro, MochoNacional, Pantaneiro, CriouloLageano do RS, CaracuCaldeano selected for milk, and Caracu selected for beef). The genomic data was used to (i) compare the overall LD decay patterns of different populations, (ii) estimate the effective population size (*N*_e_) across generations, (iii) evaluate ROH across populations, and (iv) evaluate the breed composition across all populations.

## Materials and Methods

### Data

Samples used in this study were obtained from Brazilian commercial herds and sent by the owners to Embrapa Research Centers, Brazil. In the case of males, semen samples were sent, and in the case of females, blood aliquots were sent from routine collection to monitor Brucellosis disease in the herds. Since biological samples from all animals were collected by the proprietors during routine zootechnical practices in their commercial herds, this procedure does not fit in the Brazilian National Council for the Control of Animal Experimentation law. In this way, an ethic statement was not required to generate the results in this manuscript. It is important to note that all the animals used in this study were chosen according to their population representativeness in each breed aiming to avoid bias due to the use of a limited number of lineages in the population. Thus, to safeguard the genetic variability of each breed, we used the principal components analysis to define the best breed representative samples for those with a high number of genotyped animals. For smaller samples, such as the locally adapted breeds, we did consider the pedigree and geographic distribution of the sampled animals. Animals were genotyped using commercial genotyping arrays Illumina Bovine HD, Bovine SNP50 Genotyping BeadChip (Illumina, San Diego, CA, United States), and GeneSeek GGP BosIndicus HD chip (Neogen, Lansing, MI, United States). A standardized set of single-nucleotide polymorphisms (SNPs) based on the overlapping of the SNPs among the chips was used as the data set to analyze each breed. SNP genotypes were excluded from the analysis when the call rate <0.95 and minor allele frequency <0.01. In addition, samples showing overall call rates <0.85 were excluded from further analysis. A total of 432 taurine, 366 indicine, 281 locally adapted, and 241 crossbred animals were analyzed after quality control (Table 1). Moreover, it is important to note that the remaining markers covered the whole bovine genome.

**TABLE 1:** Number of animals by breed, number of SNPs after quality control, and number of animals by group in 19 cattle breeds raised in Brazil.

Breed origin	Breed	Animals	Markers	Sample size
Taurine	ANG	31	41580	432
	BWS	34	39780	
	JER	260	35269	
	HOL	107	38946	
Indicine	BRA	30	31284	366
	GIR	127	24062	
	IND	45	28776	
	NEL	23	69448	
	SIN	92	32264	
	TAB	49	69808	
Locally adapted	CCD	55	37108	281
	CCB	24	36353	
	CRI	62	40796	
	CUR	17	30332	
	FRA	54	35444	
	MOC	20	38266	
	PAN	49	36641	
Synthetic crossbreds	CAN	121	37174	241
	GIO	120	47295	
			Total	1320

*ANG, Angus; BWS, Brown Swiss; JER, Jersey; HOL, Holstein; BRA, Brahman; GIR, Gir; IND, Indubrasil; NEL, Nelore; SIN, Sindhi; TAB, Tabapuã; CCD, Caracu Dairy; CCB, Caracu Beef; CRI, CriouloLageano; CUR, CurraleiroPé-Duro; FRA, CriouloLageano do RS; MOC, MochoNacional; PAN, Pantaneiro; CAN, Canchim; GIO, Girolando.*

### Linkage Disequilibrium and Its Decay Over Time

To evaluate the extent of LD, we used the r2 statistics, since this approach is robust and not sensitive to gene frequency and effective population size (Terwilliger et al., 2002; Zhao et al., 2007). PLINK v1.07 (Purcell et al., 2007) was used to calculate pair-wise linkage disequilibrium between SNPs using a genomic distance of 1 Mb by the following command:./plink –bfile filename –cow –ld-window 5 –ld-window-kb 1000 –ld-window-r2 0 –not-chr 0 30-33 –out outname –r2.

Parametric nonlinear regression models have been used to study the physical distance between markers (LD decay) as reported by Heifetz et al. (2005), Amaral et al. (2008), Abasht et al. (2009), and Veroneze et al. (2014). Thus, the pairwise $r_{\text{ij}}^{2}$ from each population were regressed on the distance between the marker pairs using a nonlinear model described by Sved (1971):

(1) $$LD_{ijk}=\frac{1}{\left(1+4\beta_{k}d_{ij}\right)e_{ijk}+1}$$

where *LD*_ijk_ was the observed $r_{\text{ij}}^{2}$ between SNPs *i* and *j* in breed *k*; *d*_ij_ was the distance in kb (kilobase pair) between SNPs *i* and *j*; ß_*k*_ was the coefficient that describes the LD decay with distance for breed k; and *e*_ijk_ was a random residual defined as *e*_ijk_*N*(*O*, *s*^2^). In summary, according to Sved (1971), smaller ß_*k*_ estimates indicate a higher extent of LD. Additionally, this model is useful under situations involving individual’s relationship through infinitely long pathways from remote ancestors and also by descent from recent ancestors, such as bottlenecks at some point in history followed by admixture with modern breeds mainly derived from semen importation.

A test for the equality of curves was based on ß_*k*_ estimates to compare the nonlinear curves in all breeds. This test allowed a statistical comparison of the LD decay parameter (β) and the visualization of clusters among breeds. Clustering was performed based on NbClust package v. 3.0 (Charrad et al., 2014) of software R (R Core Team), and groups were identified based on Euclidian distance under Ward aggregation method. These indices were proposed according to the choice of the best grouping based on the maximum and minimum differences, respectively, between and within group indices. Based on $\hat{ß}$ values clustering, a dendrogram was generated using the hclust function on R software v. 4.0.0 (R Core Team) in order to point out for similarities among the contrasting groups. The nonlinear models were fitted using the nls function of R software (R Core Team).

### Effective Population Size

The effective population size (*N*_e_) in the corresponding generation (T) was estimated from the associated estimated average of r^2^. Thus, *N*_e_ was estimated across generation according to the following formula:

(2) $$N_{e}=\left(\frac{1}{4c}\right)\left(\frac{1}{{\bar{r}}^{2}}-1\right)$$

where *c* is the marker distance in Morgan, assuming 1 Mb = 1 cM, and T = 1/2c (Hayes et al., 2003; Meuwissen and Goddard, 2007). From the estimated r^2^, the average values were grouped into four ranges of distances (<0.01 Mb, 0.011–0.03 Mb, 0.031–0.05 Mb, and >0.05 Mb) and used to determine the *N*_e_ in 50, 16.6, 10, and 5 generations, respectively.

### Runs of Homozygosity

To date, studies have used different methodologies for predicting ROH. Peripolli et al. (2017) evaluated several ROH studies in livestock and found that there is little consensus on the parameters used to define ROH. In this way, we established the parameters and thresholds after several “pilot” analyses as showed by Peripolli et al. (2017). The software PLINK v1.07 (Purcell et al., 2007) was used for each population with the following parameters: minimum window length of 120 SNPs, maximum gap size between two SNPs of 1,000 kb, minimum ROH length of 1,000 kb, minimum number of potential marked SNPs of 50, one heterozygote allowed per window, maximum of five missing calls per window, sliding window length of 50 SNPs, and proportion of overlapping windows that must be a homozygous >0.05.

In addition, to identify ROH islands throughout the genome of each breed, homozygous segments shared by more than 50% of the individuals were chosen as an indication of homozygosity. The “–homozyg-group” function implemented in PLINK was used to assess ROH islands shared among individuals. The GenBank annotation based on the ARS-UCD1.2 assembly of the bovine genome was used to identify genes in ROH regions. Gene networks highlighting biological processes among the gene sets identified for each breed group (taurine, indicine, locally adapted, and synthetic crossbreds) were generated using the ClueGO plugin for Cytoscape (Bindea et al., 2009). ClueGO takes one or more set of genes and pursues for Gene Ontology (GO) term or pathways establishing edges between each gene and GO chosen term using a two-sided hypergeometric test and Bonferroni correction. This procedure allowed the creation of gene networks highlighting biological roles and the comparison of gene clusters by visualizing their functional differences or similarities.

### Breed Composition

The ADMIXTURE package (Alexander et al., 2009), which places individuals into K predefined clusters based on genotype data, was used to evaluate the hierarchical clustering across the 19 evaluated breeds. Ancestry was estimated using a reduced panel (4,547 SNP) pruned by LD between subsequent markers and randomly selected animals composing reduced groups of up to 33 animals per breed. Considering our predefined breed origins (taurine, indicine, synthetic breed, and locally adapted), we evaluated all populations by using K values ranging from 2 to 5. To investigate the phylogenetic relationships among populations, the Euclidean distance between populations was evaluated using dartR package (Gruber et al., 2018) in R, and then, a phylogenetic tree was constructed using a neighbor-joining approach with APE program (Paradis et al., 2004). The principal component analysis (PCA) was performed with SNPs data using the Variation Suite Golden Helix v8.9.0 software (Golden Helix Inc., Bozeman, MT, United States).

## Results

### Linkage Disequilibrium

**FIGURE 1:**
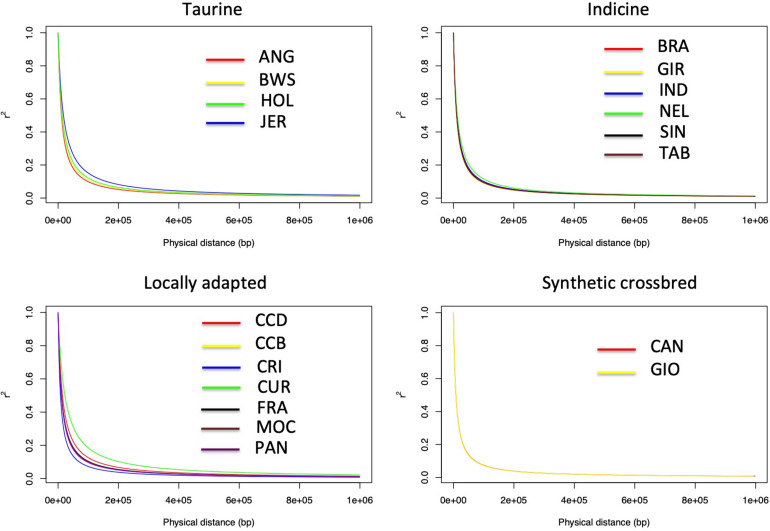
Linkage disequilibrium decay of 19 cattle breeds raised in Brazil. Linkage disequilibrium decay illustrating the r^2^ values across physical distance in base pairs (bp) of the bovine genome in the synthetic, taurine, indicine, and locally adapted groups. ANG, Angus; BWS, Brown Swiss; JER, Jersey; HOL, Holstein; BRA, Brahman; GIR, Gir; IND, Indubrasil; NEL, Nelore; SIN, Sindhi; TAB, Tabapuã; CCD, Caracu Dairy; CCB, Caracu Beef; CRI, CriouloLageano; CUR, CurraleiroPé-Duro; FRA, CriouloLageano do RS; MOC, MochoNacional; PAN, Pantaneiro: CAN, Canchim; GIO, Girolando.

**FIGURE 2:**
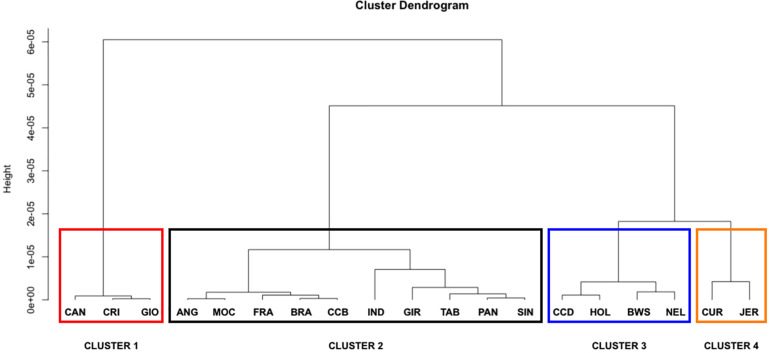
Clustering of 19 cattle breeds raised in Brazil. Dendrogram showing population clusters based on LD decay using Euclidian distance and Ward’s aggregation method. ANG, Angus; BWS, Brown Swiss; JER, Jersey; HOL, Holstein; BRA, Brahman; GIR, Gir; IND, Indubrasil; NEL, Nelore; SIN, Sindhi; TAB, Tabapuã; CCD, Caracu Dairy; CCB, Caracu Beef; CRI, CriouloLageano; CUR, CurraleiroPé-Duro; FRA, CriouloLageano do RS; MOC, MochoNacional; PAN, Pantaneiro; CAN, Canchim; GIO, Girolando.

The LD decay parameter (β) was estimated from the curves of r^2^ values over genomic distances (bp) from each population using a nonlinear regression model (Table 2). Statistical tests for the equality of LD decay curves showed that the overall pattern of the inflection point differed among breeds of taurine, indicine, and locally adapted groups, except for synthetic crossbreds (Figure 1). Since smaller values of $\hat{ß}$ indicate a higher extent of LD, it was used for clustering the different breeds accordingly to these estimates (Figure 2). Based on that, we were able to observe four distinct clusters. CAN, CRI, and GIO (cluster 1) showed the smallest extent of LD values, while CUR and JER (cluster 4) showed the highest extent of LD values.

**TABLE 2:** LD decay parameter estimates ($\hat{ß}$) and standard errors (SE) with *p* < 10^–6^ as the significance of β parameter of Sved model fitted to Brazilian cattle breeds.

Breed	$\hat{\text{ß}}$	SE
GIO: Girolando	3.211 × 10^–5^	2.4640 × 10^–2^
CRI: CriouloLageano	3.193 × 10^–5^	2.2346 × 10^–2^
CAN: Canchim	3.150 × 10^–5^	2.2845 × 10^–2^
IND: Indubrasil	2.780 × 10^–5^	2.8360 × 10^–2^
GIR: Gir	2.544 × 10^–5^	2.8594 × 10^–2^
TAB: Tabapuã	2.441 × 10^–5^	4.2235 × 10^–2^
SIN: Sindhi	2.375 × 10^–5^	3.9809 × 10^–2^
PAN: Pantaneiro	2.344 × 10^–5^	3.3130 × 10^–2^
ANG: Angus	2.277 × 10^–5^	3.4902 × 10^–2^
BRA: Brahman	2.277 × 10^–5^	3.8608 × 10^–2^
MOC: MochoNacional	2.259 × 10^–5^	3.1316 × 10^–2^
CCB: Caracu (Beef)	2.205 × 10^–5^	3.1080 × 10^–2^
FRA: Crioulo Lageano do RS	2.153 × 10^–5^	3.5613 × 10^–2^
BWS: Brown Swiss	2.038 × 10^–5^	3.3468 × 10^–2^
NEL: Nelore	1.907 × 10^–5^	5.2770 × 10^–2^
HOL: Holstein	1.812 × 10^–5^	4.0766 × 10^–2^
CCD: CaracuCaldeano (Dairy)	1.734 × 10^–5^	4.3804 × 10^–2^
JER: Jersey	1.401 × 10^–5^	5.6142 × 10^–2^
CUR: CurraleiroPé-Duro	1.102 × 10^–5^	6.2879 × 10^–2^

### Effective Population Size

**FIGURE 3:**
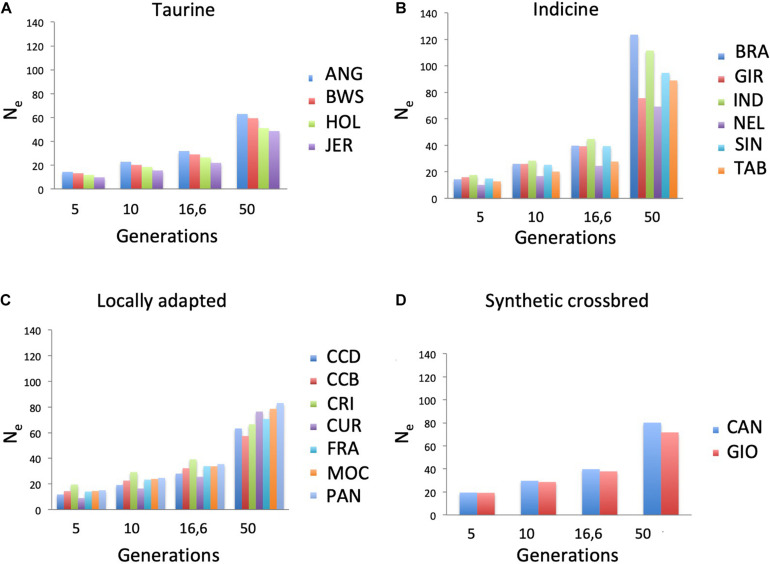
Effective population size (*N*_e_) across four generations in nineteen cattle breeds raised in Brazil. Breeds are separated into **(A)** taurine, **(B)** indicine, **(C)** locally adapted, or **(D)** synthetic crossbred groups. ANG, Angus; BWS, Brown Swiss; JER, Jersey; HOL, Holstein; BRA, Brahman; GIR, Gir; IND, Indubrasil; NEL, Nelore; SIN, Sindhi; TAB, Tabapuã; CCD, Caracu Dairy; CCB, Caracu Beef; CRI, CriouloLageano; CUR, CurraleiroPé-Duro; FRA, CriouloLageano do RS; MOC, MochoNacional; PAN, Pantaneiro: CAN, Canchim; GIO, Girolando.

*N*_e_ was estimated across four generations classes (5, 10, 16.6, and 50) according to markers distances in the genome, in which the shorter is the marker distance, the higher is the estimated generation (Figure 3). To observe the behavior of *N*_e_ across generations in each population, the difference between generations 50 and 5 was calculated (Supplementary Table 1). BRA showed the largest difference in *N*_e_ values, ranging from 123.53 at generation 50 to 14.3 at generation 5. On the other hand, JER showed the smallest values, ranging from 48.55 to 9.79 at generation 50 and 5, respectively. CUR breed showed the smallest *N*_e_ value (9.02) at generation 5.

### Runs of Homozygosity

**FIGURE 4:**
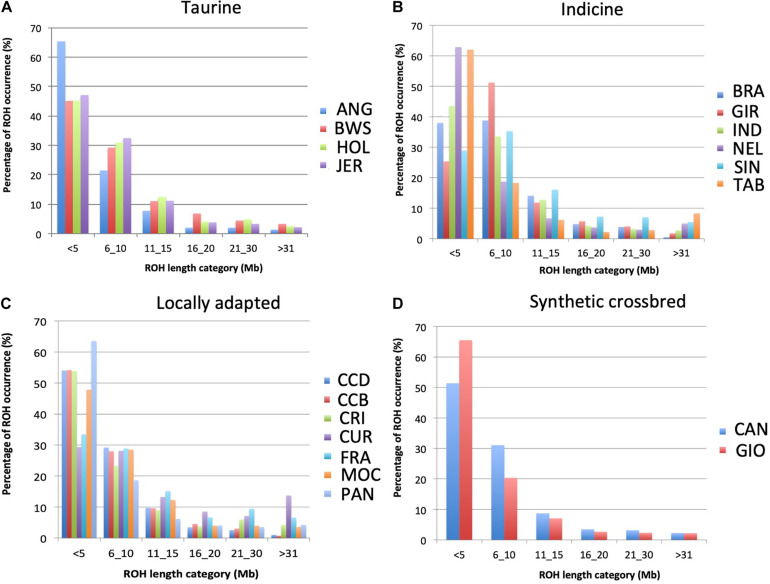
Percentage of runs of homozygosity (ROH) per length categories in 19 cattle breeds raised in Brazil. The sum of ROH was calculated per animal within each ROH length category for 19 breeds separated into **(A)** taurine, **(B)** indicine, **(C)** locally adapted, and **(D)** synthetic crossbreds groups. ANG, Angus; BWS, Brown Swiss; JER, Jersey; HOL, Holstein; BRA, Brahman; GIR, Gir; IND, Indubrasil; NEL, Nelore; SIN, Sindhi; TAB, Tabapuã; CCD, Caracu dairy; CCBO, Caracu beef; CRI, CriouloLageano; CUR, CurraleiroPé-Duro; FRA, CriouloLageano do RS; MOC, MochoNacional; PAN, Pantaneiro; CAN, Canchim; GIO, Girolando.

The pattern of ROH differed across breeds (Figure 4). Considering all breeds, CUR showed the highest percentage of long-range ROH (> 31Mb; >10%), while ANG, NEL, TAB, GIO, and PAN showed the higher percentages of short range ROH (<5 Mb; >60%). In addition, based on ROH islands analysis, seven regions were observed to be shared by more than 50% of the individuals in JER (taurine); BRA, IND, and TAB (indicine); CCD, CCB, and CUR (locally adapted) breed groups (Table 3). Gene networks highlighting biological associations were generated based on genes found on ROH islands (Supplementary Figures 1–3). Several biological processes were highlighted, including neutrophil-mediated killing of bacteria, interferon-alpha response, and prolactin signaling pathway.

**TABLE 3:** Chromosomal position, length, number of markers, and gene content of runs of homozygosity (ROH) islands shared by more than 50% (ROH freq.) identified in taurine (JER), indicine (BRA, IND, and TAB), and locally adapted (CCD, CCB, and CUR) breeds.

Breed origin	Breed	Chr.	Begin	End	Length (bp)	No. SNP	ROH freq. (%)	Annotated genes
Taurine	JER	7	42,096,163	43,741,249	1,645,086	17	68	*OR2T27, OR2T2, LOC788259, OR2T11, OR2G6, LOC510631, LOC788323, LOC787015, LOC507971, LOC509922, OR2T4, LOC112447667, LOC524160, LOC615281, LOC517722, LOC518474, LOC100299275, MGC137030, LYPD8, LOC112447409, SH3BP5L, ZNF672, ZNF692, TRNAL-CAA, TRNAE-CUC, LOC100139823, PGBD2, LOC104972393, LOC789031, LOC789041, LOC789049, LOC614592, LOC785149, LOC100300085, LOC112447517, LOC112447518, LOC112447519, LOC522560, LOC788709, LOC112447520, LOC787611, VN2R410P, LOC112447521, PLPP2, MIER2, THEG, C2CD4C, SHC2, ODF3L2, MADCAM1, TPGS1, LOC112447550, CDC34, GZMM, BSG, HCN2, POLRMT, TRNAE-UUC, FGF22, RNF126, FSTL3, PRSS57, PALM, MISP, PTBP1, PLPPR3, LOC112447411, AZU1, LOC112447659, PRTN3, ELANE, CFD, MED16, LOC112447653, R3HDM4, KISS1R, ARID3A, WDR18, GRIN3B, TMEM259, CNN2, ABCA7, ARHGAP45, POLR2E, GPX4, SBNO2, STK11, CBARP, ATP5F1D, MIDN, CIRBP, C7H19orf24, EFNA2*
Indicine	BRA	14	23,498,304	23,964,115	465,811	9	63	*SDR16C6, PENK, LOC112449660, IMPAD1*
	IND	6	36,614,641	40,780,850	4,166,209	35	58	*PKD2, SPP1, LOC112447053, MEPE, IBSP, LOC112447150, TRNAA-CGC, LAP3, MED28, FAM184B, NCAPG, DCAF16, LCORL, LOC782905, SLIT2, LOC100138737, MIR218-1, PACRGL, KCNIP4*
	TAB	1	2,210,624	3,033,433	823	30	55	*IFNAR1, LOC104970778, IL10RB, IFNAR2, LOC112447121, LOC526226, LOC107132172, OLIG1, LOC100848368, OLIG2, LOC112447133, C1H21orf62, PAXBP1, SYNJ1, LOC107132171, LOC112447136, CFAP298, LOC112447137, EVA1C*
Locally adapted	CCD	20	38,452,484	39,965,197	1,512,713	21	69	*SPEF2, PRLR, AGXT2, DNAJC21, BRIX1, RAD1, TTC23L, LOC112443042, LOC112443006, RAI14, LOC107131570, C1QTNF3, LOC112443059, AMACR, SLC45A2, RXFP3, ADAMTS12*
	CCB	20	38,108,826	38,675,350	566,524	9	79	*SKP2, LMBRD2, UGT3A2, LOC525484, CAPSL, IL7R, SPEF2*
	CUR	11	54,590,248	60,271,069	5,680,821	57	59	*CTNNA2, LOC783021, LOC112448911, LRRTM1, LOC112448799, LOC112448800, LOC107132934, REG3G, REG3A, LOC112448971, LOC524703, TRNAC-GCA, LOC100139625, LOC100138942, LOC112448942, LRRTM4, LOC101907748, LOC784779, LOC112448801, C11H2orf74, AHSA2, USP34, LOC101908099, TRNAG-CCC, XPO1*

*JER, Jersey; BRA, Brahman; IND, Indubrasil; TAB, Tabapuã; CCD, Caracu Dairy; CCB, Caracu Beef; CUR, CurraleiroPé-Duro.*

### Breed Composition

**FIGURE 5:**
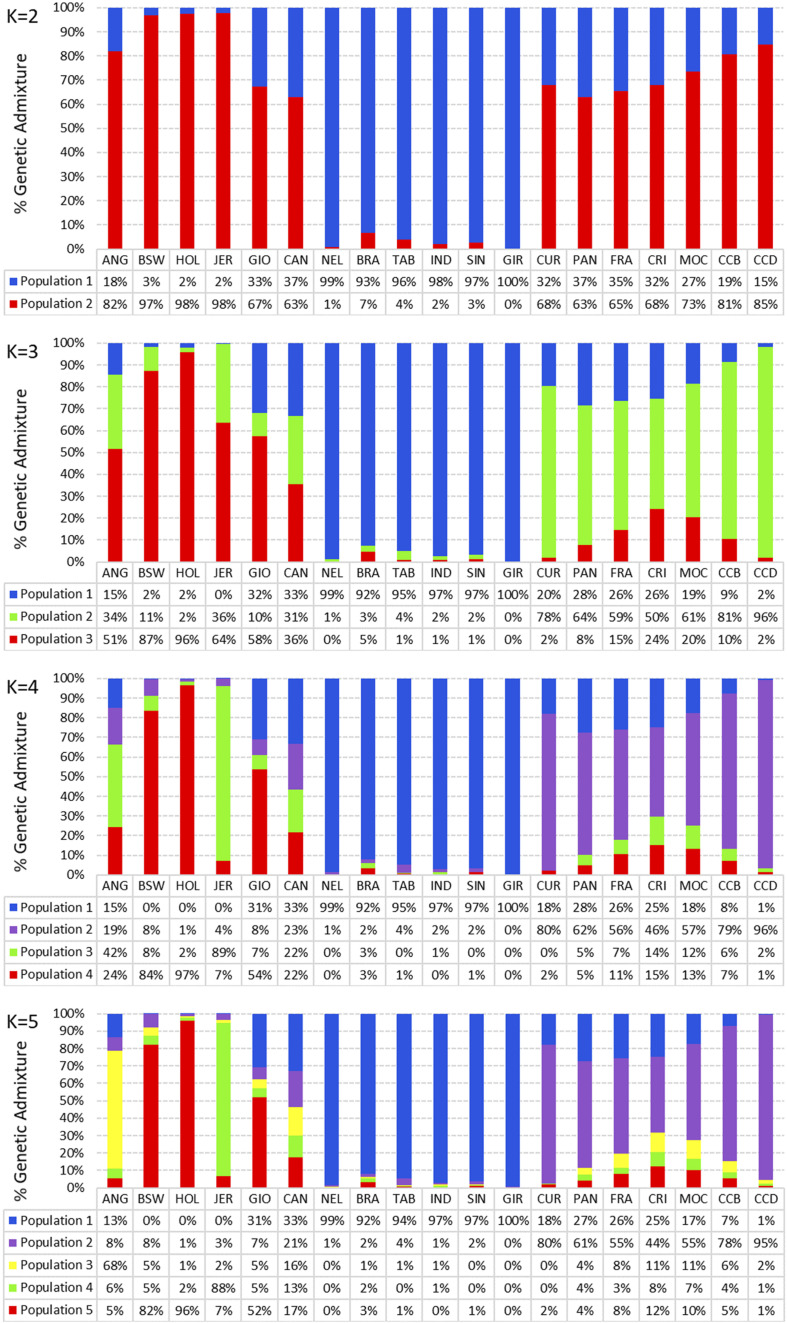
Breed composition of nineteen cattle breeds raised in Brazil. Admixture analysis using the first 5 K solutions for 19 populations grouped by taurine, synthetic crossbreed, indicine, and locally adapted animals. ANG, Angus; BWS, Brown Swiss; JER, Jersey; HOL, Holstein; BRA, Brahman; GIR, Gir; IND, Indubrasil; NEL, Nelore; SIN, Sindhi; TAB, Tabapuã; CCD, Caracu Dairy; CCB, Caracu Beef; CRI, CriouloLageano; CUR, CurraleiroPé-Duro; FRA, CriouloLageano do RS; MOC, MochoNacional; PAN, Pantaneiro; CAN, Canchim; GIO, Girolando.

Breed composition for each population was obtained using the admixture analysis (Figure 5). With *K* = 2, we observed that each group showed distinct genomic compositions representative of taurine (red) and indicine breeds (blue). Locally adapted breeds display a taurine genome content ≥65%. In addition, it was consistently shown that synthetic crossbreds shared taurine and indicine genome content. At *K* = 3, two taurine ancestries were discriminated (light green and red), whereas at *K* = 4, a third taurine ancestry was predominant in the locally adapted breeds (purple), and the light green taurine ancestry was predominant in the JER breed. At *K* = 5, ANG breed showed a distinct taurine ancestry (yellow), whereas CAN displayed four uniform taurine composition (red, green, yellow, and purple). At this K, we could notice four taurine breeds showing three well-defined taurine ancestry (yellow, 68%; red, >82%; and light green, 88%). GIO breed showed the red taurine ancestry of 52%, while in CAN breed, the taurine ancestry was shared by the red (17%), green (13%), yellow (16%), and purple (21%) components. Among the indicine breeds, we observed a well-defined indicine ancestry in blue (>92%), whereas NEL and GIR breeds showed the highest ancestries (99% and 100%, respectively). The locally adapted breeds showed mostly the purple taurine ancestry (>43%), and CCD was the most homogeneous (95%).

**FIGURE 6:**
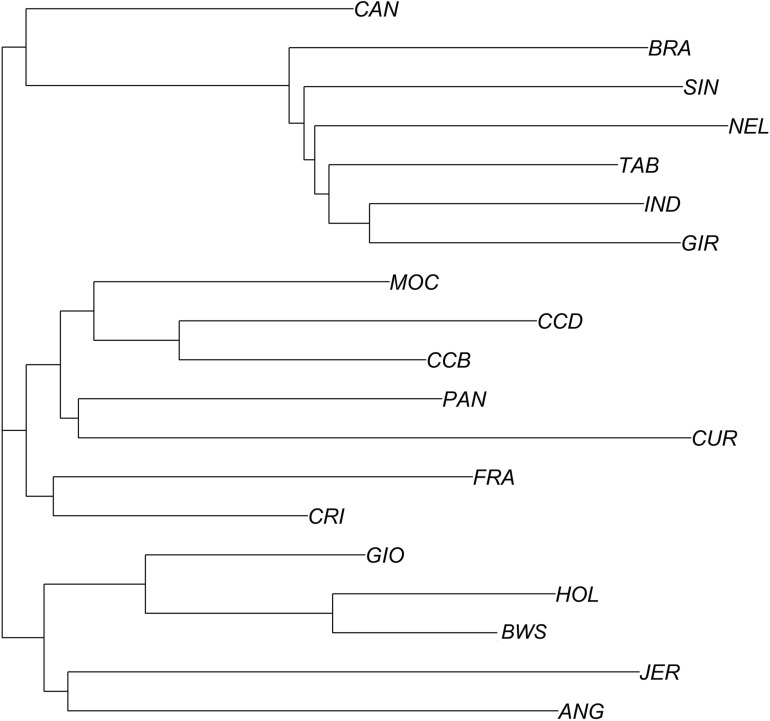
Phylogenic tree showing relationships between 19 cattle breeds raised in Brazil. Phylogenetic tree constructed using a neighbor-joining approach using Euclidian genetic distance. Breeds are labeled as Canchim (CAN), Brahman (BRA), Sindhi (SIN), Nelore (NEL), Tabapuã (TAB), Indubrasil (IND), Gir (GIR), MochoNacional (MOC), Caracu Dairy (CCD), Caracu Beef (CCB), Pantaneiro (PAN), CurraleiroPé-Duro (CUR), CriouloLageano do RS (FRA), CriouloLageano (CRI), Girolando (GIO), Holstein (HOL), Brown Swiss (BWS), Jersey (JER), Angus (ANG).

**FIGURE 7:**
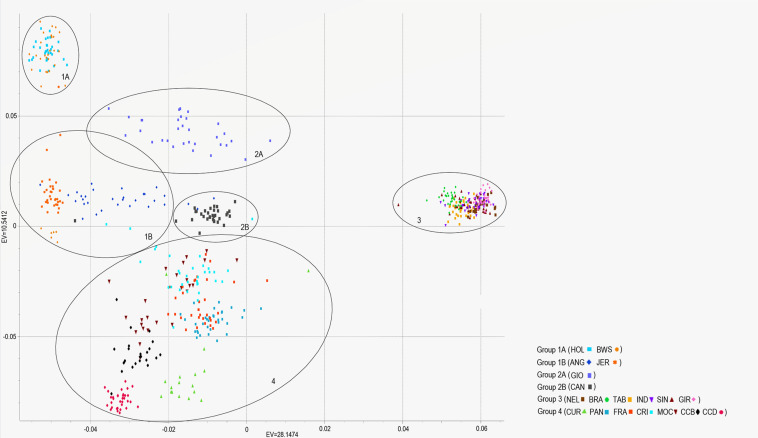
PCAs showing the relationships between nineteen cattle breeds raised in Brazil. Breeds are separated into six groups (oval circles), being taurine (groups 1A and B), synthetic crossbred (groups 2A and B), indicine (group 3), and locally adapted (group 4). Breeds are labeled as Canchim (CAN), Brahman (BRA), Sindhi (SIN), Nelore (NEL), Tabapuã (TAB), Indubrasil (IND), Gir (GIR), MochoNacional (MOC), Caracu Dairy (CCD), Caracu Beef (CCB), Pantaneiro (PAN), CurraleiroPé-Duro (CUR), CriouloLageano do RS (FRA), CriouloLageano (CRI), Girolando (GIO), Holstein (HOL), Brown Swiss (BWS), Jersey (JER), and Angus (ANG).

In addition, the neighbor-joining analysis also clearly discriminated the history of development and the ancestry of each group of breeds (Figure 6). As expected, indicine, taurine, and locally adapted breeds showed very distinct and separated clustering. The crossbred GIO was grouped with taurine breeds closer to HOL, while CAN grouped with the indicine breeds closer to BRA. The PCAs (Figure 7) were able to bring more details in the clustering pattern of the breeds and showed six distinct groups. The indicine breeds clustered very close together (group 3), and the taurine breeds showed two distinct clusters: group 1A (HOL and BWS) and group 1B (ANG and JER). The locally adapted breeds clustered together in group 4, while the crossbred GIO clustered in group 2A, and the synthetic CAN clustered in group 2B.

## Discussion

The LD decay observed in this study among all cattle breeds differed based on LD curves parameter estimates ($\hat{ß}$). However, all breeds followed a similar pattern of rapid decrease in LD as the physical distance increases in the genome. Previous LD decay studies in different breeds were performed using the average r^2^ and showed the same pattern of LD decrease (McKay et al., 2007; Lu et al., 2012; Zhu et al., 2013; O’Brien et al., 2014; Mokry et al., 2014; Toro Ospina et al., 2019; Xu et al., 2019), although they did not use a linear model to analyze the equality of the curves as performed in this study. We were able not only to observe the existence of breed differences but also do a pairwise comparison across all breeds. Based on this test, we were able to identify four LD decay clusters.

The extent of LD provides an insight about the number of SNPs required for GS and GWAS. Breeds that clustered together showed the same LD decay pattern, suggesting that the same marker density could be used in genomic studies for each of these breeds. However, this does not imply that the same marker set is suitable for a particular breed cluster group since different markers may be segregating in different breeds. For example, in cluster 3, we observed the presence of taurine and indicine breeds (HOL and NEL, respectively) highlighting the similarity of LD extent, although they have different genetic backgrounds. Thus, the set of SNPs that provides the best prediction accuracy at a given marker density should be different between HOL and NEL. We also observed different extents of LD between clusters 1 and 4, with higher LD observed in cluster 4 and lower in cluster 1. This information implies that different marker densities may be used for GS and GWAS for these breeds, which could also influence the accuracy of GS. However, in a practical way, a minimal marker density of 60 kb would be useful for all breeds besides removing monomorphic SNPs.

A run of homozygosity is the probability that all consecutive markers on a pair of homologous chromosome segments, in the same or different individual(s), display identical alleles (Hayes et al., 2003). The extent and frequency of ROH may provide information about the ancestry of an individual and its population. Even thought different threshold values could be implied from different results for the same population, our aim was to compare breeds according to their ROH. For that, we used the same parameters and thresholds for all breeds, and we were able to observe relevant different patterns of ROH among them. Moreover, inbreeding may be inferred from the presence of long ROH, with longer segments indicating recent inbreeding within a population (Kirin et al., 2010) and suggesting a small effective population size. Thus, we also evaluated the *N*_e_ to better understand the relationship between ROH and *N*_e_. The CUR breed showed the lowest *N*_e_ value in the estimated generation 5, suggesting a recent inbreeding that is supported by the occurrence of the longest ROH found in this study. Using microsatellite markers, Egito et al. (2007) also detected inbreeding in this breed, which is a locally adapted breed originated from taurine animals brought to Brazil during the first decades of colonization. According to Carvalho (2015), this breed comprises approximately five thousand individuals only. Based on our results, special attention should be given to matting schemes in this breed to preserve the germplasm.

On the other hand, further locally adapted breeds such as Caracu (CCB and CCD), CRI, and PAN seem to be successful in relation to matting schemes, since these breeds showed higher *N*_e_ and prevalence of small ROH. As expected, synthetic crossbreds (GIO and CAN) showed high occurrence of small ROH and *N*_e_, since these breeds were recently originated from a systematic crossing breeding up to the establishment of each breed. The BRA breed showed the largest differences in *N*_e_ values across generations, 123.53 at generation 50 to 14.3 at generation 5, suggesting strong inbreeding events along generations that could be explained by the breeding programs that presumably minimized the inbreeding occurrence in this breed.

JER, BRA, IND, TAB, CCD, CCB, and CUR breeds showed ROH islands in more than 50% of the individuals. From those islands, genes were retrieved and their biological processes analyzed aiming to understand their roles in each breed. In JER, we identified genes in chromosome 7 associated with lipid translocation (*ABCA7*) and neutrophil-mediated killing of bacteria (*AZU1* and *ELANE*). *ABCA7* gene encodes for ATP binding cassette subfamily A member 7 protein, previously reported to be expressed in the mammary gland of lactating cows (Farke et al., 2008) and related with lipid transport (Ontsouka and Albrecht, 2014). *AZU1* and *ELANE* encode for azurocidin 1 and elastase, neutrophil expressed proteins, respectively, which are associated with the immune system (Cassatella et al., 2019). These results corroborate the fact that Jersey animals are known to exhibit less disease (e.g., clinical mastitis) (Washburn et al., 2002) and higher fat content in milk (Carroll et al., 2006).

ROH islands were observed on chromosome 14, 6, and 1 in the indicine breeds BRA, IND, and TAB, respectively. In the homozygous regions of BRA, we found a gene related to behavioral fear response (*PENK*). It is reported that animals with Brahman genetics may be considered more reactive to humans when compared with taurine breeds, such as Angus and Hereford (Boissy et al., 2005). In the IND ROH islands, we found the positive regulation of estradiol secretion biological process associated with SPP1 gene that encodes for secreted phosphoprotein 1, in which variations have been associated with lactation persistency in dairy cattle (Bissonnette, 2018) and mammary health status (Alain et al., 2009). We found *IFNAR1* and *IFNAR2* genes, associated with response to interferon-alpha biological process, in the chromosome 1 of TAB breed. These genes have been reported to be expressed in bovine placentome tissues during early pregnancy, associated with immune tolerance protection of the mother and fetus (Wang et al., 2018). Indicine cattle are well known by their rusticity, so the presence of ROH islands linked to genes associated with the immune system and health status was expected.

We also observed ROH islands in locally adapted breeds CCD, CCB, and CUR. Interestingly, CCD and CCB shared a small segment of their islands on chromosome 20, in which the *SPEF2* gene was found. This gene encodes for sperm flagellar 2 protein, which is suggested to play an important role in elongating spermatids to ensure proper male germ cell differentiation in mice (Lehti et al., 2017). In addition, homozygosis mutations in this gene have been reported to be associated with sperm morphological abnormalities and male infertility in humans (Liu et al., 2020). Based on that, this gene could be a potential candidate for male fertility in cattle and should be further studied.

Furthermore, from the genes found in ROH islands of CCD breed, we found the prolactin receptor gene (*PRLR*), which was found to be associated with the prolactin signaling pathway biological process. Likewise, variations in this gene have been associated with milk performance traits (Zhang et al., 2008; Lü et al., 2011). On the other hand, a highlighted biological process detected in the CCB breed was the negative regulation of T-cell-mediated cytotoxicity, associated with *IL7R* gene. In humans, variations in this gene have been linked with autoimmune risk (Al-Mossawi et al., 2019), while in livestock animals, it has been described to be associated with growth traits (Jia et al., 2019; Zhu et al., 2019), which corroborates the purpose of this breed formation as a beef cattle.

In the locally adapted CUR breed, we found an ROH island on chromosome 11, and the biological process network highlighted a subnetwork involved with negative regulation of JAK-STAT cascade, linked to *LRRTM1* and *LRRTM4* genes. JAK-STAT cascade is critical to cellular events such as immune development (Rawlings et al., 2004). In addition, *LRRTM1* and *LRRTM4* genes encode for leucine-rich repeat transmembrane neuronal 1 and 4, respectively. It is suggested that leucine-rich repeat proteins family is functionally related to multiple diseases in humans (Winther and Walmod, 2014). Considering the known adaptability and rusticity of CUR breed and the immune system-related genes identified in this study, we could suggest that CUR breed has been successfully selected for rusticity regardless of its small population effective size found in this study.

The breed composition for each population was obtained using the admixture model, neighbor-joining analysis, and PCAs. In this study, we evaluated the proportion of genotypes across the 19 breeds by altering the K value from 2 up to 5. At a *K* = 2, distinct genotypes representative of taurine and indicine breeds were observed. The taurine ANG breed showed what seems to be a composition from indicine background (18%). A reasonable explanation for that may be the use of indicine animals for cross breeding or another population founder effects. Indicine breeds were used in the beginning of their production in Brazil aiming to obtain adapted animals for the tropical weather (Madalena et al., 2002). This is supported by the ROH results from this study, in which a great occurrence of short ROH was observed for ANG breed, in agreement with the crossbreeding hypothesis. Distribution of shorter ROH may also suggest the presence of more ancient kinship, which is unaccounted in pedigree records due to the limitations in the extension of the recording processes (McQuillan et al., 2008). At *K* = 2, the locally adapted breeds displayed a taurine genome content ≥ 65%. This was expected since these breeds were brought to South America brought by European colonizers and were mainly formed by taurine breeds along their history.

At *K* = 3, two taurine genotypes were discriminated, whereas in *K* = 4, a third taurine ancestry could be found in the locally adapted breeds. This genetic background noticed in the adapted animals may be the result of the Iberian origin of these animals. At K = 4, a light green taurine ancestry was highlighted in the JER breed. This could be explained by the fact that this breed was only “discovered” around 200 years ago in the Jersey island, and this fact could have contributed to the maintenance of a conserved and distinct genetic composition of this breed, especially when compared with HOL, as also observed by Huson et al. (2020).

Moreover, the *K* = 5 highlighted the four taurine breeds showing three distinct taurine ancestries (ANG, yellow; BWS and HOL, red; and JER, light green) in addition to the supposed Iberian-derived taurine composition (purple) observed in locally adapted animals. This scenario is in agreement with cattle domestication events that occurred in Europe, in which European breeds would descend from domestication events of aurochs (*Bosprimigenius*) in the Near East and its further introduction throughout the European continent. Environmental conditions and domestication events could explain the diverse taurine backgrounds found in Europe. In this way, southern European breeds could have been more affected by introductions from northern Africa as well (Beja-Pereira et al., 2006).

At *K* = 5, the GIO breed showed mainly the red taurine composition (52%), while in the CAN breed, the taurine composition was shared by red, green, yellow, and purple taurine ancestries. GIO breed was developed for milk production in tropical and subtropical conditions through HOL with GIR breeds crossing followed by mattings schemes to establish the genetic composition of 5/8 HOL and 3/8 GIR (Silva et al., 2017). On the other hand, CAN breed was developed to generate beef cattle with higher growth rate and meat quality adapted to the tropical environment. CAN genetic background was formed by crossing taurine Charolais breed with indicine Guzerat, IND, and NEL breeds, with mattings being directed to set the genetic composition of 5/8 Charolais and 3/8 indicine (Barbosa, 2000).

At K = 5, we noticed the presence of a highly homogeneous indicine ancestry in the indicine breeds BRA, GIR, IND, NEL, SIN, and TAB. Although the importation of indicine breeds started approximately 150 years ago and have been intensively genetically improved in the last 40–50 years, we highlight that the indicine background is very conserved, especially in NEL and GIR breeds that showed ≥99% of indicine background. This information could be used to support international animal market between Brazil and foreign countries such as India and other tropical cattle producer countries that were recently interested in importing this indicine-improved breeds.

As expected, the locally adapted breeds CCB, CRI, CUR, FRA, MOC, and PAN showed a mostly taurine and mainly purple ancestry background, but also displayed indicine background ranging from 7 to 27%. The indicine background may have been incorporated due to recent crossing with indicine breeds in Brazil. One exception was the CCD breed that showed 95% of the purple taurine and only 1% of indicine backgrounds. Caracu lineages (dairy and beef) were originated by crossings between breeds brought from Iberian Peninsula to Brazil, including the Portuguese Minhota Breed. Natural selection throughout centuries enabled their adaptation to adverse conditions, contributing to their rusticity and high feed efficiency (3). In addition, these breeds were kept as purebred in regions of São Paulo state for meat production (CCB) and selected in Minas Gerais state for milk production (CCD), originating these two Caracu lineages. In a previous study of genetic diversity with locally adapted and zebu breeds, we also observed that CCD showed a homogeneous population (Campos et al., 2017), corroborating with the higher taurine background found in this study.

Moreover, our results of neighbor-joining analysis and PCAs reinforce the admixture results in the genetic discrimination among cattle breeds and their development history. From the neighbor-joining analysis, indicine, and taurine breeds are shown widely apart following the genetic distances. On the other hand, locally adapted breeds showed a closer genetic distance to taurine breeds, as predictable. The GIO breed clustered with taurine breeds and closer to HOL, while CAN clustered with the indicine breeds and closer to BRA breed, as expected. The PCAs also showed the same results overall, and highlighted the genetic proximity of the indicine breeds, as they clustered together in a close and narrowed span group. Thus, it is possible to assume that indicine breeds have kept their majority genetic background from their ancestors in India, although they have been extensively selected and genetically improved for important economical traits in Brazil.

In this study, we adjusted a linear model to analyze the equality of the LD curves across 19 cattle breeds raised in Brazil, which revealed different extents of LD. Our results suggest that different marker densities may be used for a giving accuracy of GS and GWAS in these breeds. We found that CUR breed showed the lowest *N*_e_ value in the estimated generation 5, suggesting a recent inbreeding also reflected by the occurrence of longest ROH islands in our analyses. Thus, a special attention in matting schemes for this breed is needed. Genes located in ROH islands were evaluated and explored throughout their biological processes, which provided genetic insights about the breeds. We were successful in revealing the breed composition of all breeds besides the complex taurine and indicine compositions in all breeds. In addition to the LD clustering results, we suggest that taurine SNP genotyping arrays may also be used in locally adapted breeds, since they seem to share most of their genetic architecture and LD extent.

A general overview of the genetic architecture of 19 cattle breeds raised in Brazil was successfully obtained. Special attention should be given to CUR and JER breeds in order to improve matting schemes regarding the low values of *N*_e_. Even though BRA breed did not show the smallest *N*_e_ in this study, it showed a significant decrease in *N*_e_, ranging from 123.53 at generation 50 to 14.3 at generation 5, and must be carefully examined by the current breeding programs. Candidate genes were found on ROH islands in various breeds, and their association with important traits were highlighted (e.g., *ABCA7* with JER milk protein content; *PENK* with behavior fear response in BRA; *SPP1* with mammary health status in IND; *IFNAR1* and *IFNAR2* with immune system features in TAB; *SPEF2* with male infertility in both CCD and CCB; and *PRLR* with milk production in CCD; *LRRTM1* and *LRRTM4* with negative regulation of JAK-STAT cascade in CUR).

## Data Availability Statement

The original contributions presented in the study are included in the article/Supplementary Material, further inquiries can be directed to the corresponding author.

## Ethics Statement

Ethical review and approval was not required for the animal study because it used genotypes data provided.

## Author Contributions

LV, MSi, FS, MM, and JC conceived, designed, and supervised the experiments. LV and MSi conducted the experiment. LV analyzed the results and prepared the manuscript draft. DL, PO, LA, LS, AE, MSo, MM, and LV edited the manuscript. All authors reviewed and approved the final manuscript.

## Conflict of Interest

The authors declare that the research was conducted in the absence of any commercial or financial relationships that could be construed as a potential conflict of interest.

## Publisher’s Note

All claims expressed in this article are solely those of the authors and do not necessarily represent those of their affiliated organizations, or those of the publisher, the editors and the reviewers. Any product that may be evaluated in this article, or claim that may be made by its manufacturer, is not guaranteed or endorsed by the publisher.
