# Supplementary material for: Genome-Wide Analyses Reveal the Genetic Architecture and Candidate Genes of Indicine, Taurine, Synthetic Crossbreds, and Locally Adapted Cattle in Brazil
Source: Front Genet. 2021 Jul 27;12:702822. doi: 10.3389/fgene.2021.702822 (PMC8353373; doi:10.3389/fgene.2021.702822)
Supplement: Supplementary file 1 [file Data_Sheet_1.docx]

Supplementary Material

## Supplementary Figures


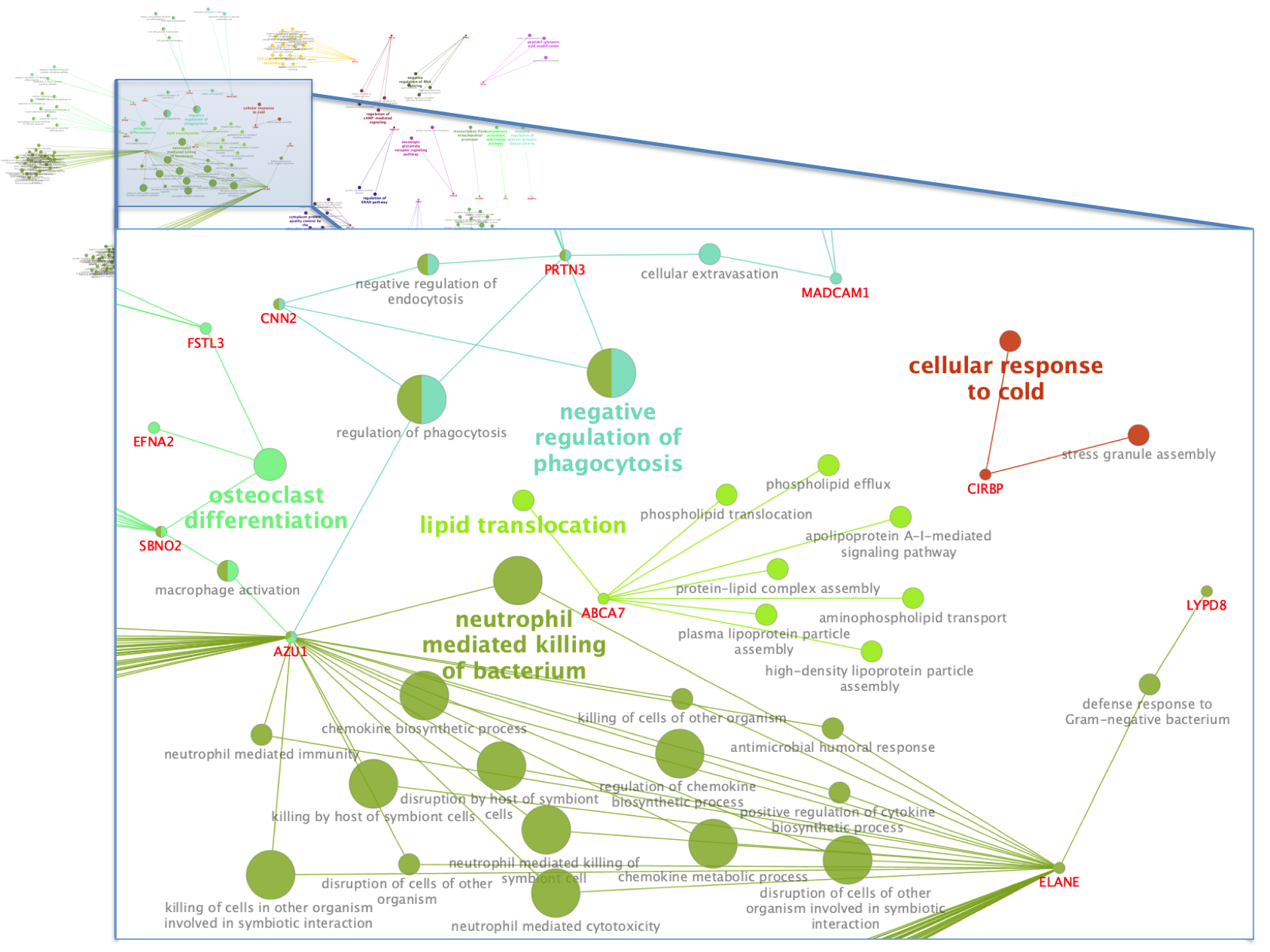


**Supplementary Figure 1.** Main functional biological process networks of genes observed inside runs of homozigosity islands identified in Angus herd. Biological process (BP) terms and genes (labeled in red) are presented as nodes. Different colors of nodes represent sub-networks of BP associated with those genes. The figure shows a zoom highlighting the main enriched terms that are shown in bold according to enrichment significance from the ClueGO Cytoscape plugin (Bindea et al., 2009).


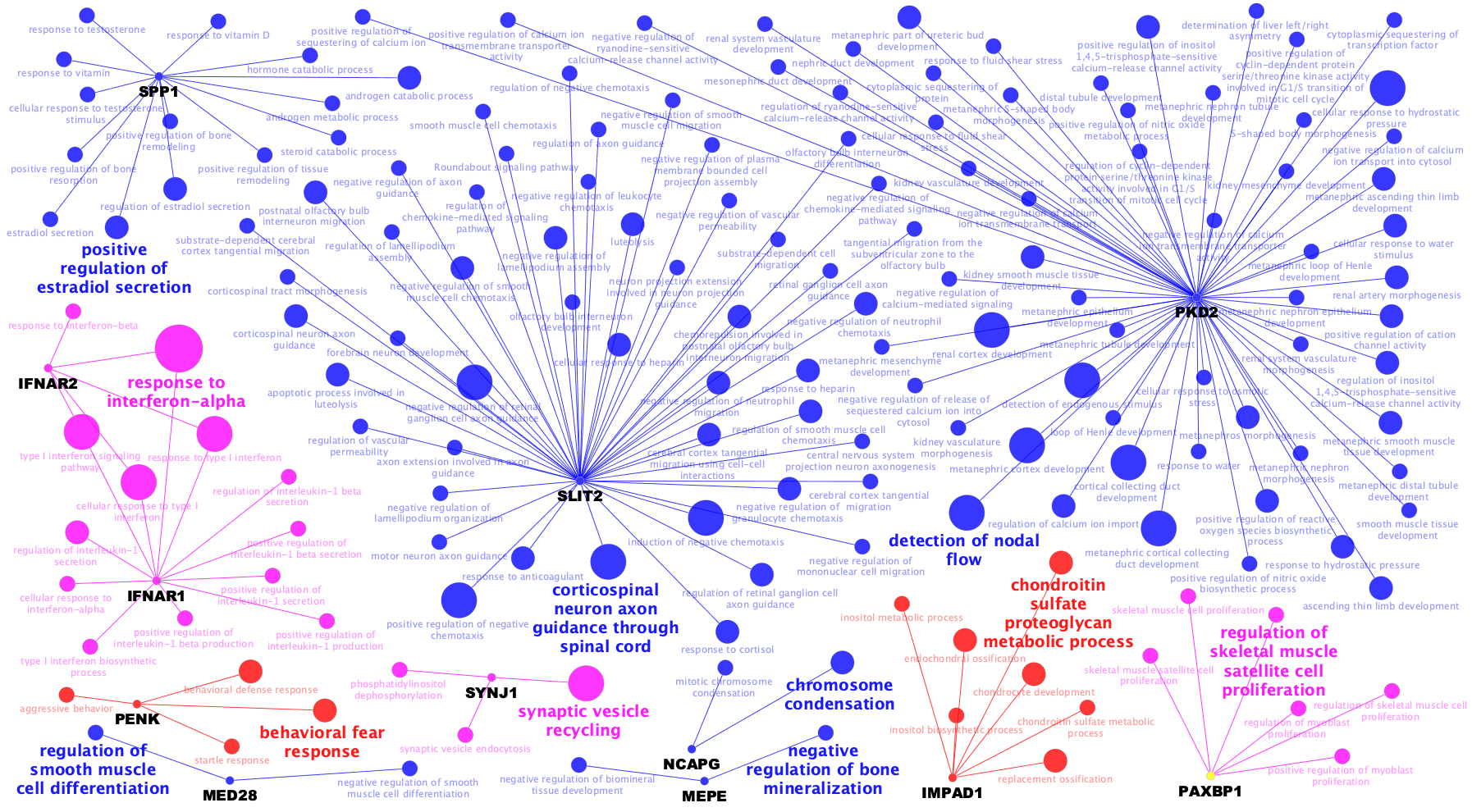


**Supplementary Figure 2.** Main functional biological process networks of genes observed inside runs of homozigosity islands identified in indicine herds. Biological process (BP) terms and genes (labeled in black) are presented as nodes. Red, blue and pink nodes are BP and genes observed in Brahman, Indubrasil and Tabapuã herds, respectively. The most enriched terms are shown in bold according to enrichment significance from the ClueGO Cytoscape plugin (Bindea et al., 2009).

**
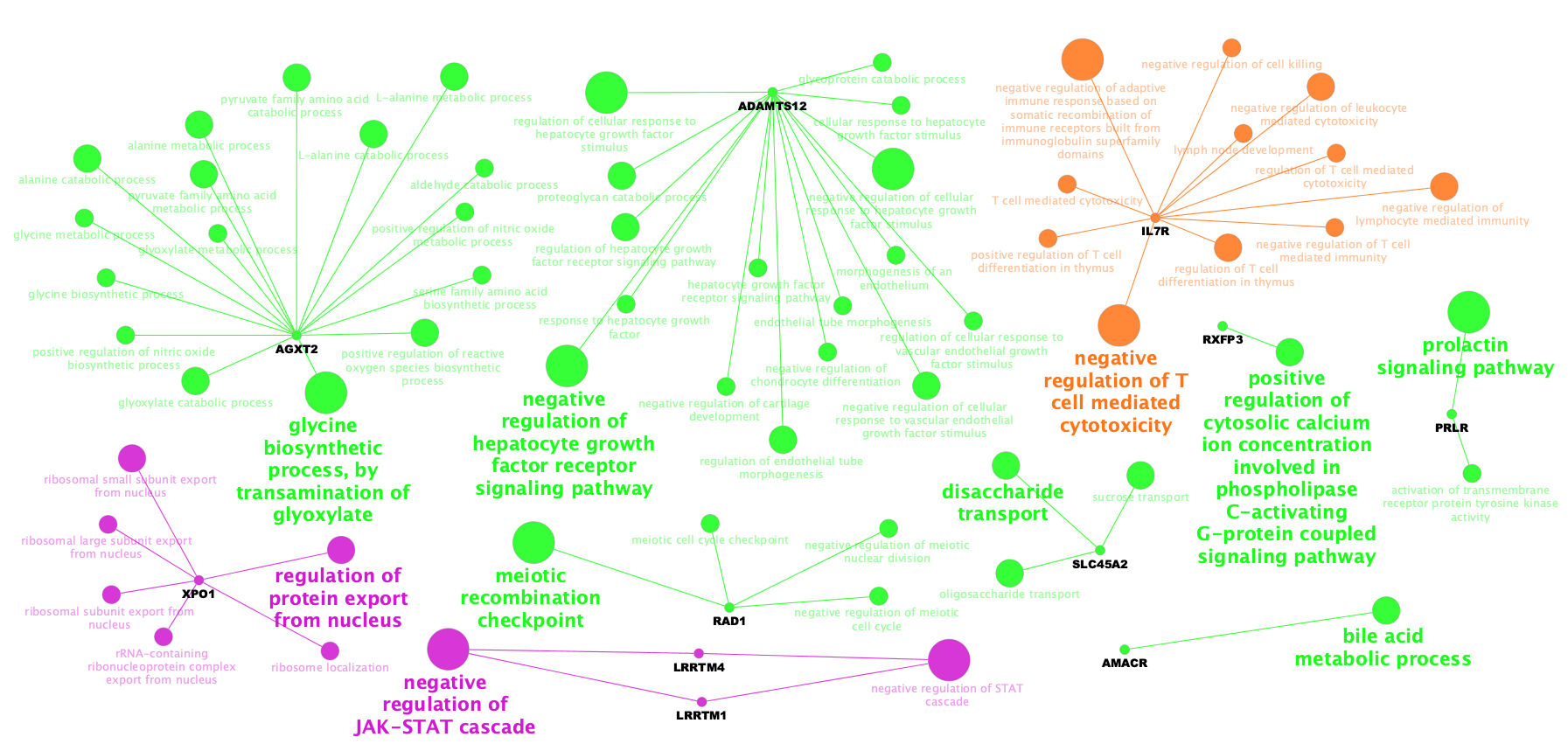
**

**Supplementary Figure 3.** Main functional biological process networks of genes observed inside runs of homozigosity islands identified in locally adapted herds. Biological process (BP) terms and genes (labeled in black) are presented as nodes. Green, orange and purple nodes are BP and genes observed in Caracu Dairy, Caracu Beef and Curraleiro Pé-Duro herds, respectively. The most enriched terms are shown in bold according to enrichment significance from the ClueGO Cytoscape plugin (Bindea et al., 2009).

**Supplementary Table**

**Table 1**. Population effective size across four generations and difference between generations 50 and 5 (Dif.) in nineteen cattle breeds raised in Brazil.

| **Breed*** | **Generations** | | | | |
| --- | --- | --- | --- | --- | --- |
|  | **5** | **10** | **16.6** | **50** | **Dif.** |
| ANG | 14.34 | 22.78 | 31.79 | 62.90 | 48.57 |
| BRA | 14.36 | 26.04 | 39.75 | 123.54 | 109.19 |
| BWS | 13.15 | 20.16 | 28.97 | 59.35 | 46.19 |
| CAN | 19.47 | 29.72 | 39.84 | 80.17 | 60.71 |
| CCD | 11.77 | 19.17 | 28.06 | 63.25 | 51.48 |
| CCB | 14.38 | 22.61 | 32.26 | 57.40 | 43.02 |
| CRI | 19.58 | 29.32 | 39.20 | 66.58 | 46.99 |
| CUR | 9.02 | 16.46 | 25.60 | 76.46 | 67.44 |
| FRA | 14.08 | 23.38 | 33.90 | 70.79 | 56.71 |
| GIR | 15.99 | 25.98 | 39.34 | 75.60 | 59.61 |
| GIO | 19.24 | 28.65 | 37.89 | 71.71 | 52.47 |
| HOL | 11.86 | 18.47 | 26.45 | 51.01 | 39.15 |
| IND | 17.66 | 28.42 | 44.88 | 111.54 | 93.88 |
| JER | 9.79 | 15.51 | 21.89 | 48.55 | 38.76 |
| MOC | 14.52 | 23.95 | 33.78 | 78.61 | 64.09 |
| NEL | 10.18 | 16.82 | 24.54 | 69.27 | 59.08 |
| PAN | 15.08 | 24.73 | 35.48 | 83.08 | 68.00 |
| SIN | 14.99 | 25.28 | 39.42 | 94.73 | 79.74 |
| TAB | 12.79 | 20.19 | 27.74 | 89.00 | 76.21 |

*ANG: Angus; BWS: Brown Swiss; JER: Jersey; HOL: Holstein; BRA: Brahman; GIR: Gir; IND: Indubrasil; NEL: Nelore; SIN: Sindhi; TAB: Tabapuã; CCD: Caracu Dairy; CCB: Caracu Beef; CRI: Crioulo Lageano; CUR: Curraleiro Pé-Duro; FRA: Crioulo Lageano do RS; MOC: Mocho Nacional; PAN: Pantaneiro: CAN: Canchim; GIO: Girolando
